# Effects of Exercise on Oxidative Stress in Rats Induced by Ozone

**DOI:** 10.1100/2012/135921

**Published:** 2012-04-24

**Authors:** Catalina Martinez-Campos, Eleazar Lara-Padilla, Rosa Amalia Bobadilla-Lugo, Robert David Kross, Cleva Villanueva

**Affiliations:** ^1^Seción de Estudios de Posgrado e Investigación, Escuela Superior de Medicina, IPN, Plan de San Luis y Salvador Diaz Mirón S/N, Colonia Casco de Santo Tomás, 11340 México, DF, Mexico; ^2^Escuela Médico Militar, Department of Morphology, Boulevard Avila Camacho y Cerrada de Palomas S/N, Colonia Lomas de Sotelo, 11640 México, DF, Mexico; ^3^Universidad Pablo de Olavide, Facultad del Deporte, Carretera de Utrera Km 1, Edificio 2, Planta Baja, 41013 Sevilla, Spain; ^4^Kross-Link Laboratories, P.O. Box 374, Bellmore, NY 11710, USA

## Abstract

Oxidative stress (OS) induced by acute exercise is reduced by chronic exercise. Ozone (O_3_) exposure produces OS. The aim of this study was to determine if aerobic exercise (AE) reduced OS produced by O_3_. A pilot experiment was performed with male Wistar rats submitted to AE (trained to swim 90 min/day). Adaptation to exercise was demonstrated three weeks after training by means of changes in reduced nitrates (NO_*x*_) in plasma. Therefore, two-week training was chosen for the following experiments. Six of twelve trained rats were exposed to O_3_ (0.5 ppm, 4 h/day, one hour before exercise). Two groups of sedentary animals (*n* = 6 each) were used as controls, one of which was exposed to O_3_. At the end of the experiments NO_*x*_, 8-isoprostane (8-IP), malondialdehyde (MDA), superoxide dismutase (SOD) activity, and carbonyls (CBs) were measured in plasma. CBs did not change in any group. O_3_-induced OS was manifested by reduced NO_*x*_ and SOD activity, as well as increased 8-IP and MDA. Exercise significantly blocked O_3_ effects although SOD was also decreased by exercise (a greater drop occurring in the O_3_ group). It is concluded that AE protects against OS produced by O_3_ and the effect is independent of SOD.

## 1. Introduction

Oxidative stress (OS) produced by acute exercise is characterized by an excess of free radicals. It was thought that mitochondria were the main source of free radicals in exercise; however, it is now known that even though mitochondria do contribute, other sources are the main contributors (xanthine oxidase, NADPH oxidase, and phospholipase A2) [1–3]. Chronic exercise reduces OS produced by acute exercise [1, 4, 5]. Adaptation to exercise is due, in part, to an increase in the endogenous antioxidant defense [1, 4–7]. The increase of nitric oxide (NO) availability takes part in the adaptation and the benefits produced by long-term exercise [8–10].

It is thought that the mechanism of adaptation to exercise includes activation of nuclear factor kappa B (NF*κ*B) by free radicals, which upregulates the synthesis of endothelial NO synthase (eNOS) and antioxidant enzymes [8, 9, 11, 12]. Long-lasting moderate exercise has benefic effects such as prevention of certain cancers, prolonged lifespan in rodents, reduction of cardiovascular effects of aging and menopause, better metabolic control and renal as well as cardiovascular protection in diabetes, and improvement of chronic heart failure [3, 5–7, 11, 13–16].

Ozone (O_3_) is a common pollutant in urban areas. The effects of O_3_ extend beyond the lung. O_3_ exposure produces systemic OS [17]. O_3_ exposure has been associated with premature mortality [18], cardiovascular mortality [19], myocardial infarction [20], and cerebrovascular diseases [21]. OS and endothelial dysfunction have been related to the cardiovascular toxic effects of O_3_ [22].

The goal of this study was to determine if moderate aerobic exercise affected OS produced by O_3_.

## 2. Material and Methods

### 2.1. Animals

Male Wistar rats, 10 weeks old (230–250 g), were supplied by Harlan Mexico. Animals were fed with Purina chow and water *ad libitum* and submitted to light/dark periods of 12/12 h. Animals were kept in a room fed with filtered air to maintain O_3_ within normal concentrations (<0.05 ppm) according to the USA Environmental Protection Agency (http://www.epa.gov/air/ozonepollution/standards.html). The local Institutional Animal Committee approved all the procedures.

### 2.2. Training Protocol

Rats were trained to swim 90 min a day, 7 days a week. Animals swam in water at 35–37°C, in groups of 6, in a pool measuring 92 cm long, 42 cm wide, and 32 cm deep. Animals were progressively trained during the first week, beginning with 15 min the first day and then increased every day by 15 min periods to reach 90 min on the sixth day. After that the animals swam continuously for 90 min per day. The exercise was performed everyday at noon. After exercise the animals were carefully dried and maintained at room temperature (25°C).

### 2.3. Ozone Exposure

O_3_ exposure was made in groups of six animals in an OTC-1 chamber (In USA, Inc.). The chamber had a servomechanism to maintain O_3_ concentrations at 0.5 ± 0.05 ppm. The chamber was programmed to destroy O_3_ in such a way that it was impossible to open the chamber if O_3_ concentration was above normal (≥0.05 ppm). Animals were exposed to O_3_ 4 hours a day, every day (07:00–11:00 h).

### 2.4. Groups

Groups of 6 animals were formed as follows.

1. Pilot groups: eight pilot groups were formed in order to analyze adaptation to exercise. Half of those groups were sedentary (kept in their cages, which allowed for free movement) and half were submitted to aerobic exercise, as mentioned above. One, two, four, or eight weeks after training, two groups (sedentary and trained) were anesthetized (sodium pentobarbital 45 mg/Kg, ip). The left carotid was cannulated with a PE50 catheter, and a blood sample (3 mL) was taken and treated with EDTA. The animals where then sacrificed by anesthesia overdose. Adaptation to exercise was evaluated by measuring reduced nitrates (NO_*x*_) in plasma using the Griess method (Cayman Chemical Co kit).
2. With the results of the pilot groups (see below), a two-week period (just before adaptation) was chosen for the following experiments.
  - Sedentary group. This group remained sedentary and it was kept in the O_3_ chamber at normal concentrations (<0.05 ppm), for 4 hours a day for 2 weeks, in order to have the same confinement stress as that the rats exposed to O_3_.
  - Sedentary group exposed to O_3_: this group remained sedentary and was exposed to O_3_ (0.5 ppm, 4 h a day) daily for 2 weeks.
  - Trained group this group was kept in the O_3_ chamber at normal concentrations (see group (a)), and one hour later they were trained as explained.
  - Trained group exposed to O_3_ this group was exposed to O_3_ (0.5, 4 h a day) daily for 2 weeks. One hour after O_3_ exposure the animals swam as described.

At the end of the two-week experiment, all animals were anesthetized and 5 mL arterial blood samples were taken. The animals were then sacrificed by anesthesia overdose.

### 2.5. Oxidative Stress Evaluation

Arterial blood samples were heparinized and centrifuged at 1200×g, 15 min at 4°C. Plasma was separated and divided into 5 aliquots of 200 *μ*L to measure

- reduced nitrates (NO_*x*_, modified Griess method, Cayman Chemical Co. Kit,)
- 8-isoprostane (8-IP, Cayman Chemical Co. ELISA kit),
- Malondialdehyde (MDA, Cayman Chemical Co. TBARS kit),
- Protein carbonyls (Cayman Chemical Co. kit),
- Total activity of superoxide dismutase (SOD, Cayman Chemical Co. kit).

### 2.6. Statistical Analysis

Data are presented as mean ± standard error of the mean (SEM) of *n* experiments. Data were analyzed using the one way ANOVA test and Tukey's multiple comparison test *post hoc* or the two-way ANOVA test and the Bonferroni test *post hoc*.

## 3. Results

### 3.1. Adaptation to Exercise

**Figure 1:**
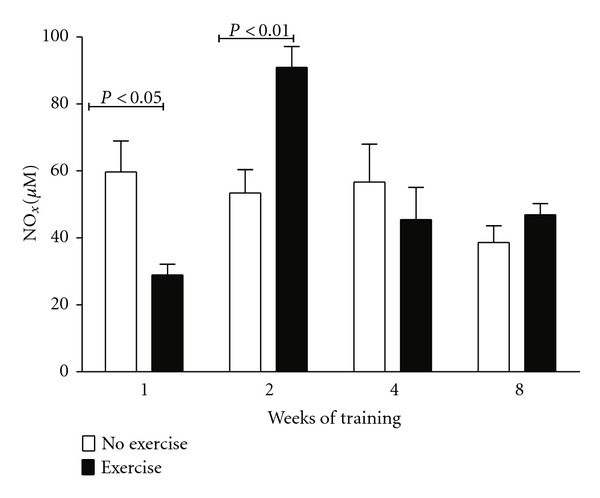
Adaptation to exercise. Plasma reduced nitrate (NO_*x*_) levels decreased significantly one week after training, whereas they significantly increased two weeks after training and returned to normal levels 4 weeks after training (with no changes thereafter). The return to normal is considered adaptation to exercise. Data were analyzed using the two-way ANOVA test and the Bonferroni test *post hoc*. Data are shown as the mean ± standard error of mean (*n* = 6 per group).

Results are shown in Figure 1. Adaptation to exercise, measured through NO_*x*_ plasma concentration, was reached after two weeks of training. Therefore, a 2-week training was chosen for the experiments where rats were or were not submitted to O_3_.

### 3.2. Oxidative Stress Measurement

**Figure 2:**
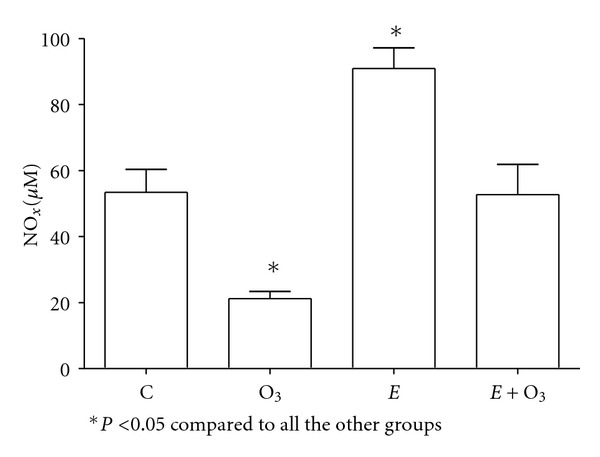
Plasma NO_*x*_ concentrations at week two. Ozone (O_3_) exposure (0.5 ppm 4 hours/day) significantly decreased whereas exercise (*E*, 90 min per day) significantly increased plasma NO_*x*_ concentrations. The effect of O_3_ exposure was completely blocked by exercise. Data were analyzed using the one-way ANOVA test and Tukey's multiple comparison test *post hoc*. Data are shown as the mean ± standard error of mean (*n* = 6 per group).

**Figure 3:**
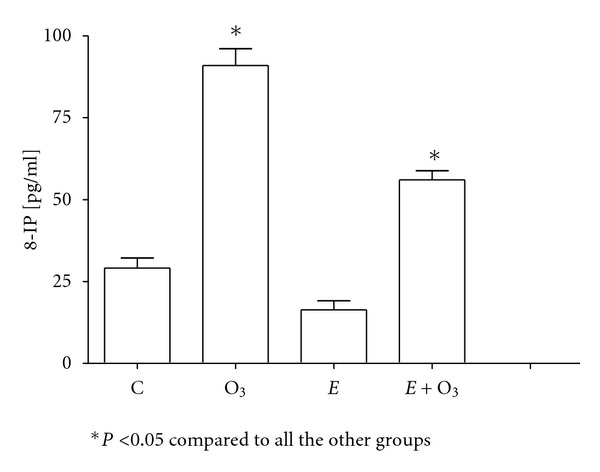
Plasma 8-isoprostane (8-IP) concentrations at week two. Ozone (O_3_, 0.5 ppm 4 hours/day) exposure significantly increased 8-IP levels. Even though exercise (90 min per day) did not change 8-IP, it partially but significantly blocked the O_3_ effect. Data were analyzed using the one-way ANOVA test and Tukey's multiple comparison test *post hoc*. Data are shown as the mean ± standard error of mean (*n* = 6 per group).

**Figure 4:**
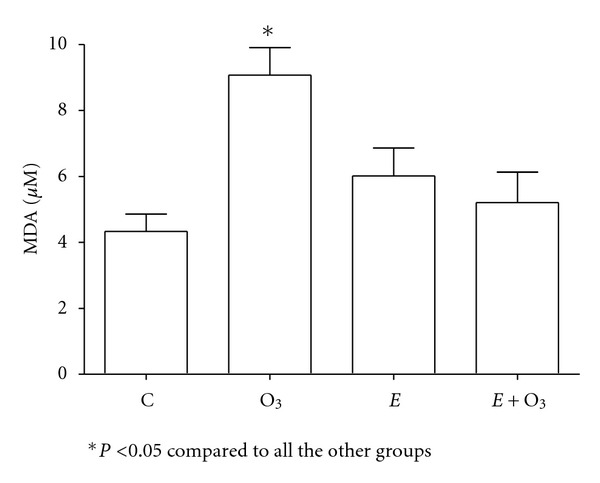
Plasma malondialdehyde (MDA) concentrations at week 2. Ozone (O_3_, 0.5 ppm 4 hours/day) exposure significantly increased MDA levels. Even though exercise (90 min per day) did not change MDA, it blocked completely O_3_ effect. Data were analyzed using the one-way ANOVA test and Tukey's multiple comparison test *post hoc*. Data are shown as the mean ± standard error of mean (*n* = 6 per group).

**Figure 5:**
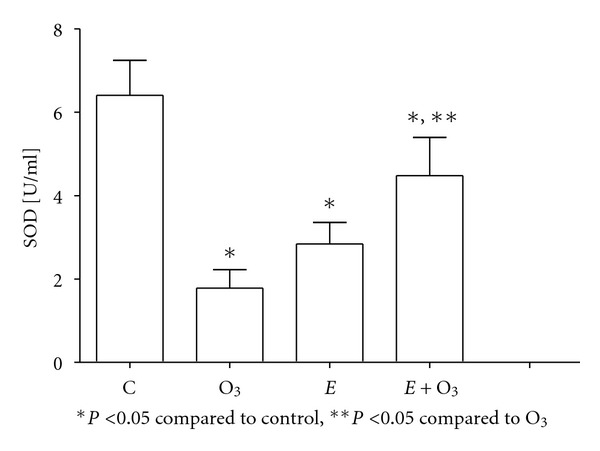
Plasma total superoxide dismutase (SOD) activity at week 2. Ozone (O_3_, 0.5 ppm 4 hours/day) exposure and exercise (*E*, 90 min per day) significantly decreased plasma SOD activity. Exercise partially blocked O_3_ effects. Data were analyzed using the one-way ANOVA test and Tukey's multiple comparison test *post hoc*. Data are shown as the mean ± standard error of mean (*n* = 6 per group).

Protein carbonyls were similar in all the groups (data not shown). O_3_ exposure significantly decreased NO_*x*_ levels (*P* < 0.05) (Figure 2), whereas it increased both 8-IP (Figure 3) and MDA levels (Figure 4) (*P* < 0.5). Exercise prevented those changes although the effect was partial on 8-IP. SOD activity (Figure 5) significantly decreased with O_3_ and independently with exercise (*P* < 0.05). However, the combination of O_3_ and exercise resulted significantly increased values of SOD activity.

## 4. Discussion

Acute exercise produces OS mainly through superoxide production [1, 2]. Chronic exercise reduces OS generated by acute exercise [1, 4, 5]. The mechanism of such adaptation seems to be through activation of NF*κ*B by free radicals, which in turn increases the synthesis of antioxidant enzymes and NO synthases [4, 9, 12, 23]. Moreover, benefits produced by exercise seem to be given, at least in part, precisely by the induction of antioxidant enzymes and NO [1, 4, 9]. In the present study adaptation to exercise, evaluated through NO production, was reached after three weeks of training. Adaptation to exercise, measured through other biomarkers, was reported previously in the same period using a similar training model [24].

Since O_3_ exposure produces OS, we wanted to know if exercise could affect such OS just before adaptation to exercise was reached. Therefore, evaluation of OS in the presence or absence of O_3_ was made with or without two weeks of training. We chose a two-week training period because it was the time when NO_*x*_ significantly increased, with no changes in concentrations thereafter.

Measurements were made in plasma in order to evaluate the systemic effects of exercise. Other authors report changes produced by exercise in skeletal muscle [24]. However, beneficial effects of exercise are probably systemic. OS produced by O_3_ was confirmed through the increase of 8-IP and MDA as well as the reduction of NO_*x*_ and SOD activity. Decreased production or OS could explain the reduction of NO. NO availability decreases during OS because it is combined with superoxide and transformed in peroxynitrite [25]. 8-IP and MAD were chosen as biomarkers of lipoperoxidation, whereas protein carbonyls were chosen as a biomarker of effects of OS on proteins. SOD was chosen because it is the first-line enzyme dealing with superoxide production.

Interestingly, two-week training protected from lipoperoxidation produced by O_3_ exposure, At the same time decreased SOD activity but partially blocked O_3_ effects on this enzyme. It was recently reported that regular exercise prevented premature mortality attributed to pollution in Chinese people older than 65 years [26]. Our findings agree with that paper and suggest that reduction of OS by chronic exercise could have a role in the protection against pollution. It is important to note that our results have the limitation of not being necessarily applicable to exercise in open environments in contact with pollution, because the animals were trained in a pool in a room with filtered air, which would be equivalent to performing indoor exercise. It would be interesting to explore OS when animals are trained in a polluted environment. It is also important to mention that these experiments were conducted with healthy animals. Results could be different in animals suffering from a disease accompanied by OS (e.g., diabetes, hypertension, and hypercholesterolemia).

SOD results are intriguing. O_3_ exposure, and independent exercise, reduced SOD activity, even though exercise with O_3_ exposure partially blocked O_3_ effects. It is known that exercise produces OS and, as a result, the endogenous antioxidant defense is increased. Indeed, it has been reported that exercise increases SOD [11, 15]. However, this effect has been observed in studies with exercise training longer than 2 weeks. Measurements of the present study were done at the end of the second week of training, just one week before adaptation to exercise. In the present study, the protection of O_3_ effects produced by exercise (effects on 8-IP, MDA, NO_*x*_, and even SOD) could be attributable to changes in other endogenous antioxidants different from SOD (e.g., glutathione peroxidase and catalase).

It is concluded that AE protects against OS produced by O_3_, and the effect is independent of SOD.
